# Analysis of risk factors related to nonalcoholic fatty liver disease: a retrospective study based on 31,718 adult Chinese individuals

**DOI:** 10.3389/fmed.2023.1168499

**Published:** 2023-06-29

**Authors:** Ganggang Wang, Xiaowei Shen, Yicun Wang, Huanhua Lu, Hua He, Xiaoliang Wang

**Affiliations:** ^1^Department of Hepatobiliary Surgery, Pudong Hospital, Fudan University, Shanghai, China; ^2^Department of General Surgery, Qingpu Branch of Zhongshan Hospital, Fudan University, Shanghai, China

**Keywords:** NAFLD, risk factors, logistic regression, ROC diagnosis, prevention

## Abstract

**Objective:**

Nonalcoholic fatty liver disease (NAFLD) is becoming increasingly prevalent worldwide. This study guides the prevention and diagnosis of NAFLD by analyzing its risk factors and the diagnostic value of each index for NAFLD.

**Method:**

We collected the clinical information of adults individuals who underwent physical examination in the Physical Examination Center of Qingpu Branch of Zhongshan Hospital, Fudan University, from January 2016 to January 2020, including gender, age, body mass index (BMI), systolic blood pressure (SBP), diastolic blood pressure (DBP), alanine aminotransferase (ALT), aspartate aminotransferase (AST), total bilirubin (TBIL), direct bilirubin (DBIL), indirect bilirubin (IBIL), fasting blood glucose (FBG), total cholesterol (TC), triglyceride (TG), high-density lipoprotein (HDL), and low-density lipoprotein (LDL). We performed logistic regression analysis and ROC diagnostic analysis.

**Results:**

The results showed that age, BMI, SBP, ALT, AST, FBG, TBIL, TG, and LDL were risk factors for NAFLD in adults, and HDL was a protective factor (all *p*-values were less than 0.05). Among them, age, BMI, ALT, TG, and HDL had a predictive value for the occurrence of NAFLD in the adults (AUC = 0.708, 0.836, 0.767, 0.780, and 0.732, respectively). The combination of age, BMI, ALT, TG, and HDL had a diagnostic value for the occurrence of NAFLD (AUC = 0.881).

**Conclusion:**

Healthy people should pay attention to their BMI levels, manage blood pressure, blood glucose, and lipid levels, and pay attention to changes in ALT and AST index levels to prevent NAFLD. Age, BMI, ALT, TG, and HDL indexes are helpful factors in the diagnosis of NAFLD.

## 1. Introduction

Non-alcoholic fatty liver disease (NAFLD), a chronic liver disease, affects approximately 1.7 billion people worldwide. The prevalence of NAFLD is estimated to be about 25% (1). In Asia, China has the highest prevalence, morbidity, and annual mortality rates of NAFLD (2). NAFLD is an umbrella term for a range of liver diseases that vary in damage severity and results in liver fibrosis, which mainly includes hepatic steatosis (NAFL) and nonalcoholic steatohepatitis (NASH) (3).

NAFLD is a progressive disease characterized by the accumulation of early liver fat (hepatic steatosis) and liver inflammation, promoting the transition from benign steatosis to more advanced NASH. Although the disease is reversible in its early stages, its treatment becomes more complex in the advanced stages. If left untreated, NASH may progress to cirrhosis, an irreversible disease state characterized by scarring of the liver tissue that may lead to HCC (4). The main causative factors closely associated with liver cancer are hepatitis B and non-alcoholic fatty liver disease. In recent years, the incidence of hepatitis B has gradually decreased owing to vaccines and enhanced hygienic practices (5). However, the incidence of NAFLD has increased to 15%, becoming the second most common liver disease after viral hepatitis (6).

Given that the majority of patients with NAFLD are predominantly asymptomatic, early diagnosis of NASH and accurate staging of fibrosis risk are critical for better stratification, monitoring, and targeted management of at-risk patients. To date, liver biopsy remains the gold standard for the diagnosis of NASH and NAFLD staging. However, its use is not widespread due to its invasive properties. In this study, we analyzed the general information, biochemical indexes, and risk factors associated with NAFLD and searched for significant, relevant diagnostic indexes from physical examination data from a population in Qingpu, Shanghai.

## 2. Materials and methods

### 2.1. General information

The study subjects underwent physical examination in the Physical Examination Center of Qingpu Branch of Zhongshan Hospital, Fudan University from January 2016 to January 2020. We collected their physical examination data. The inclusion criteria were patients diagnosed with fatty liver by B-ultrasound results, those with complete physical examination information, and patients aged ≥ 18 years. The exclusion criteria are viral hepatitis, drug-induced hepatitis, autoimmune liver disease, Wilson disease (hepatolenticular degeneration), liver cirrhosis, liver cancer, severe malnutrition, infection, bile duct infection, severe cardiovascular and cerebrovascular diseases, other metabolic or immune diseases, cachexia and other malignant tumors, and long-term alcohol intake exceeding the standard (male ≥ 20 g/d, female ≥ 10 g/d). Duplicate samples were deleted.

The patients were divided into a NAFLD group and a non-NAFLD (NO-NAFLD) group according to the criteria. Age, BMI, SBP, DBP, ALT, AST, TBIL, DBIL, IBIL, FBG, TC, TG, HDL, and LDL indexes were collected for comparison between the two groups. The diagnosis of fatty liver is based on the 2010 criteria of the Chinese Medical Association Society of Liver Diseases (6). Fatty liver can be diagnosed by abdominal ultrasound examination with two or more of the following abnormalities: (1) enhanced near-field echogenicity and diminished far-field echogenicity of the liver; (2) echogenicity of the liver parenchyma denser than that of the kidney parenchyma; and (3) poorly visualized intrahepatic vascular and biliary structures. A subsequent medical history review of patients with fatty liver was performed, and we confirmed the diagnosis of NAFLD in patients who satisfied the following criteria. (1) No history of alcohol consumption or alcohol consumption equivalent to less than 20 g of ethanol per day and less than 10 g per day in women. (2) Excluding viral hepatitis, drug-related liver disease, Wilson’s disease, total parenteral nutrition, autoimmune liver disease, and other specific diseases that can cause fatty liver. (3) Histological manifestations of the liver meet the pathological diagnostic criteria of fatty liver disease. This study was approved by the Ethics Committee of the Qingpu Branch of Zhongshan Hospital, Fudan University.

### 2.2. Statistical analysis

Data were statistically analyzed using IBM SPSS 26.0 software, and R software (version 3.6.3) was employed to visualize and graph the results of independent factors. Descriptive information was expressed as ($\bar{x}\pm s$) and analyzed via the independent samples t-test. Comparisons of categorical information between groups were made using the χ^2^ test. Data for skewed distributions were expressed as medians and quartiles and compared using the Mann–Whitney U test. When the results of the descriptive analysis were statistically different, the factor was regressed by binary logistic regression. *p* < 0.05 was considered a statistically significant difference.

One-way logistic regression analysis was performed for the indicators that had differences in the descriptive statistical results. Multi-factor logistic regression analysis was performed for indicators that differed from the one-way logistic regression analysis. Female was used as the reference gender, and NO-NAFLD was used as a reference for the rest of the indexes. After one-way logistic regression analysis, age, SBP, DBP, BMI, ALT, AST, TBIL, IBIL, FBG, TC, TG, HDL, and LDL indexes were subjected to multi-factor logistic regression analysis.

## 3. Results

### 3.1. Comparison of baseline information

A total of 31,718 physical examiners’ data were collected according to the above criteria, including 15,628 males and 16,090 females. In the 31,718-person sample, there were 16,968 patients with NAFLD and 14,750 people without NAFLD. There were differences in age, SBP, DBP, BMI, ALT, AST, TBIL, IBIL, FBG, TC, TG, HDL, and LDL between the NAFLD group and NO-NAFLD group (Table 1; *p* < 0.05).

**Table 1:** General information and biochemical detection indexes of the two groups.

Characteristic	NAFLD	NO-NAFLD	X^2^/t	*p*
Gender, *n* (%)			2127.844	0.658
Female	10,409 (32.8%)	5,219 (16.5%)		
Male	6,559 (20.7%)	9,531 (30.0%)		
Age	56.43 ± 15.358	47.12 ± 17.122	50.683	**0.000**
SBP/mmHg	136.79 ± 49.616	122.93 ± 32.107	29.061	**0.000**
DBP/mmHg	83.640 ± 10.798	77.130 ± 13.005	48.061	**0.000**
BMI	25.812 ± 2.958	21.969 ± 2.783	119.128	**0.000**
ALT/(U·L^−1^)	28.550 ± 24.454	16.310 ± 13.982	55.588	**0.000**
AST/(U·L^−1^)	25.010 ± 13.723	20.300 ± 9.504	35.932	**0.000**
TBIL/(μmol·L^−1^)	14.023 ± 5.535	13.741 ± 5.408	4.566	**0.000**
DBIL/(μmol·L^−1^)	3.918 ± 1.716	3.915 ± 1.718	0.166	0.868
IBIL/(μmol·L^−1^)	10.105 ± 4.227	9.827 ± 4.057	5.976	**0.000**
FBG/(mmol·L^−1^)	6.018 ± 1.651	5.310 ± 1.063	45.969	**0.000**
TC/(mmol·L^−1^)	5.181 ± 0.999	4.927 ± 0.931	23.376	**0.000**
TG/(mmol·L^−1^)	2.433 ± 2.065	1.318 ± 0.944	63.140	**0.000**
HDL/(mmol·L^−1^)	1.206 ± 0.266	1.446 ± 0.326	−71.079	**0.000**
LDL/(mmol·L^−1^)	3.082 ± 0.852	2.855 ± 0.783	24.750	**0.000**

The bolded text in the table indicates a significance level of *p* < 0.05.

### 3.2. Logistic regression analysis results

**Figure 1:**
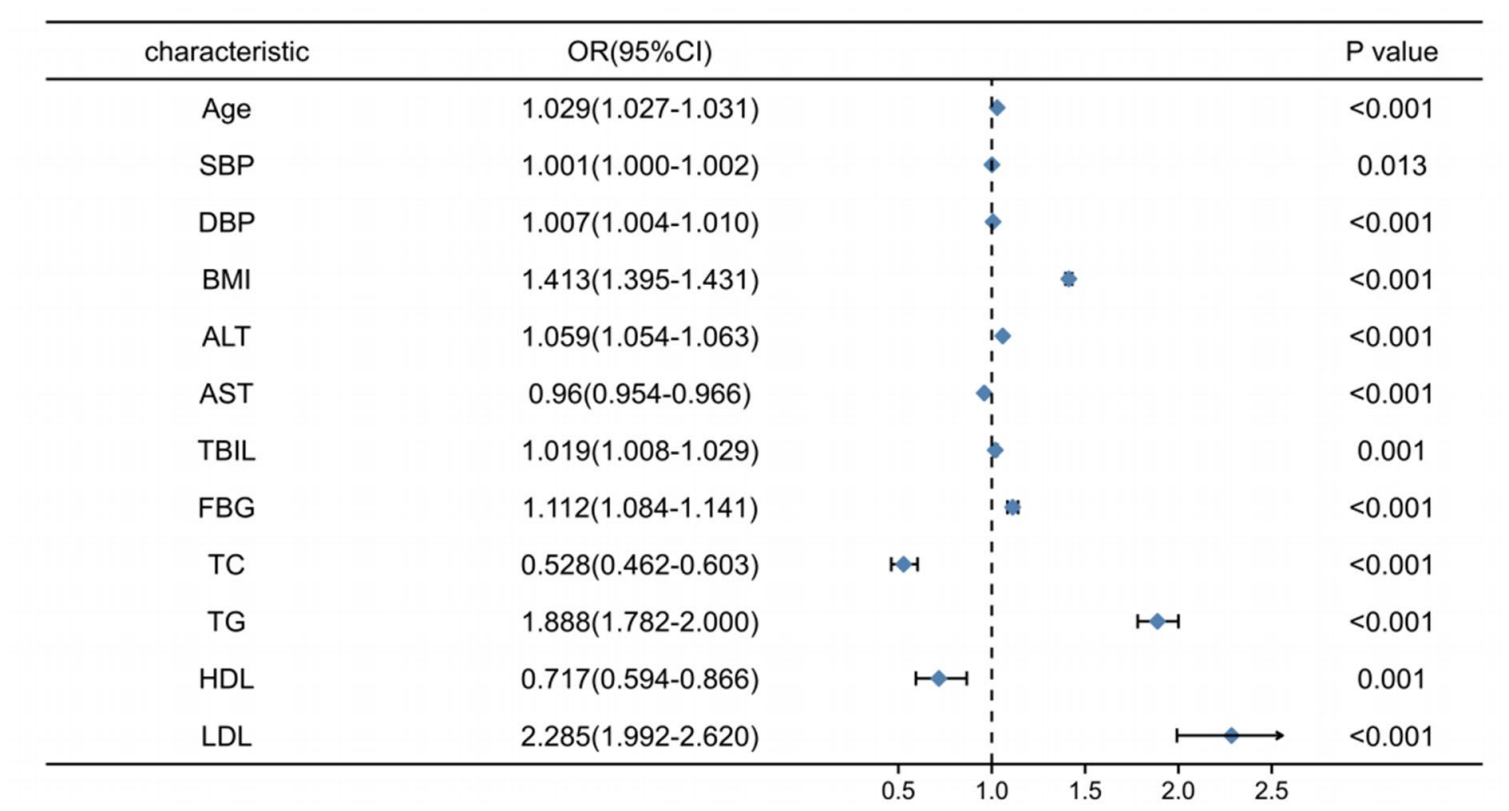
Forest plot of the difference indicators for multi-factor logistic regression analysis between the NAFLD and NO-NAFLD groups.

The final multi-factor logistic regression results showed that age, SBP, DBP, BMI, ALT, TBIL, FBG, TG, and LDL may be risk factors for the occurrence of NAFLD. AST, TC, and HDL may be protective factors for adult patients with NAFLD (Table 2; Figure 1), which indicated that obese and hypertensive patients were more likely to have NAFLD in combination.

**Table 2:** Results of univariate and multivariate logistic regression analysis of indicators of differences between the two populations.

Characteristics	Univariate analysis		Multivariate analysis	
	Odds ratio (95% CI)	*P-*value	Odds ratio (95% CI)	*P-*value
Age	1.035 (1.034–1.036)	0.000	1.029 (1.027–1.031)	0.000
SBP	1.039 (1.038–1.040)	0.000	1.001 (1.000–1.002)	0.013
DBP	1.055 (1.053–1.057)	0.000	1.007 (1.004–1.010)	0.000
BMI	1.642 (1.624–1.661)	0.000	1.413 (1.395–1.431)	0.000
ALT	1.08 (1.077–1.083)	0.000	1.059 (1.054–1.063)	0.000
AST	1.069 (1.066–1.073)	0.000	0.96 (0.954–0.966)	0.000
TBIL	1.009 (1.005–1.014)	0.000	1.019 (1.008–1.029)	0.001
IBIL	1.016 (1.011–1.022)	0.000	0.968 (0.934–1.003)	0.074
FBG	1.714 (1.669–1.759)	0.000	1.112 (1.084–1.141)	0.000
TC	1.316 (1.285–1.347)	0.000	0.528 (0.462–0.603)	0.000
TG	2.856 (2.764–2.952)	0.000	1.888 (1.782–2.000)	0.000
HDL	0.061 (0.056–0.066)	0.000	0.717 (0.594–0.866)	0.001
LDL	1.406 (1.368–1.446)	0.000	2.285 (1.992–2.620)	0.000

### 3.3. Diagnostic ROC analysis results

**Figure 2:**
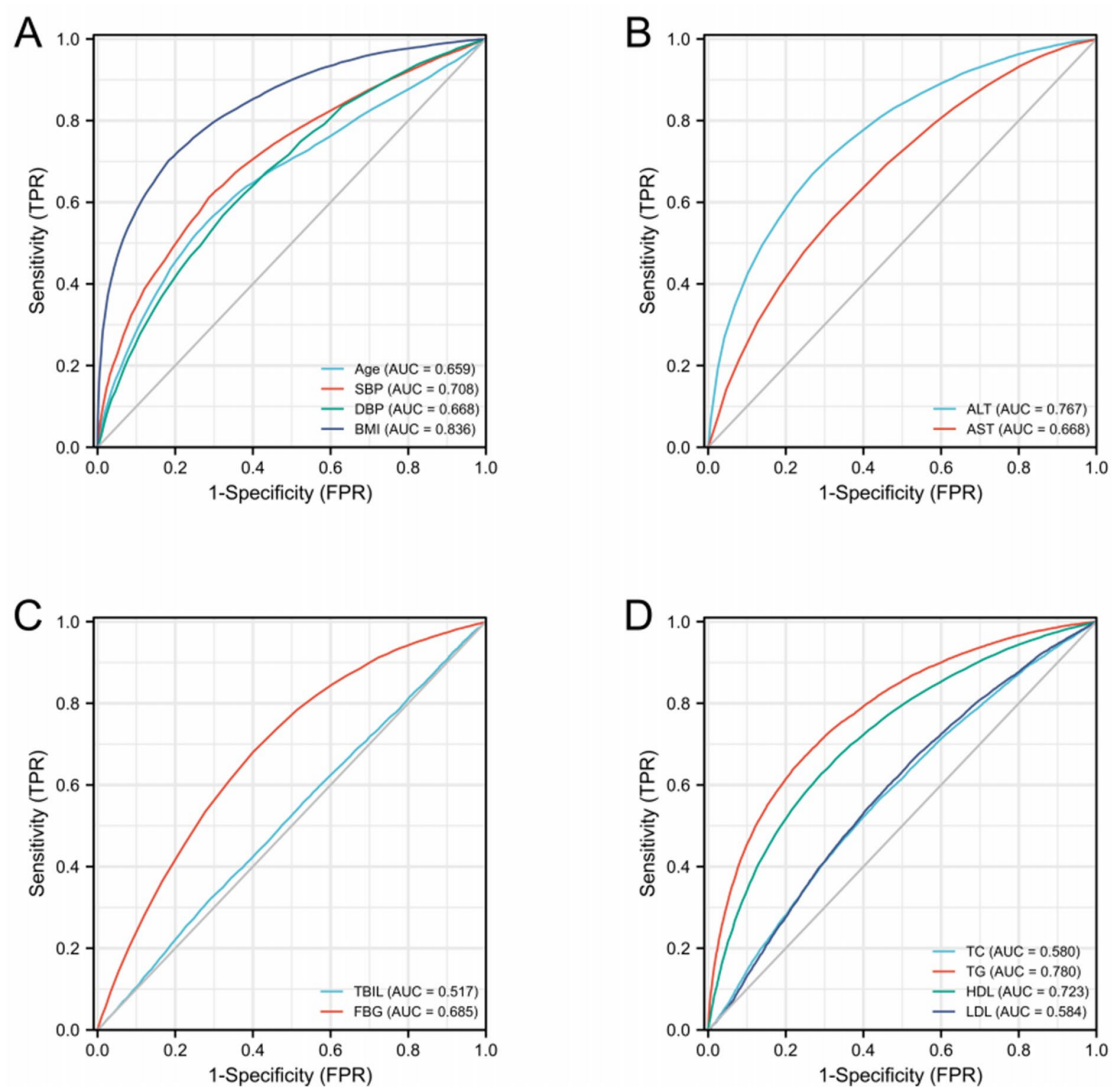
ROC curves related to differential indicators for the NAFLD and NO-NAFLD groups by multifactorial logistic regression analysis. **(A–D)** ROC curves of different discriminatory indicators.

**Figure 3:**
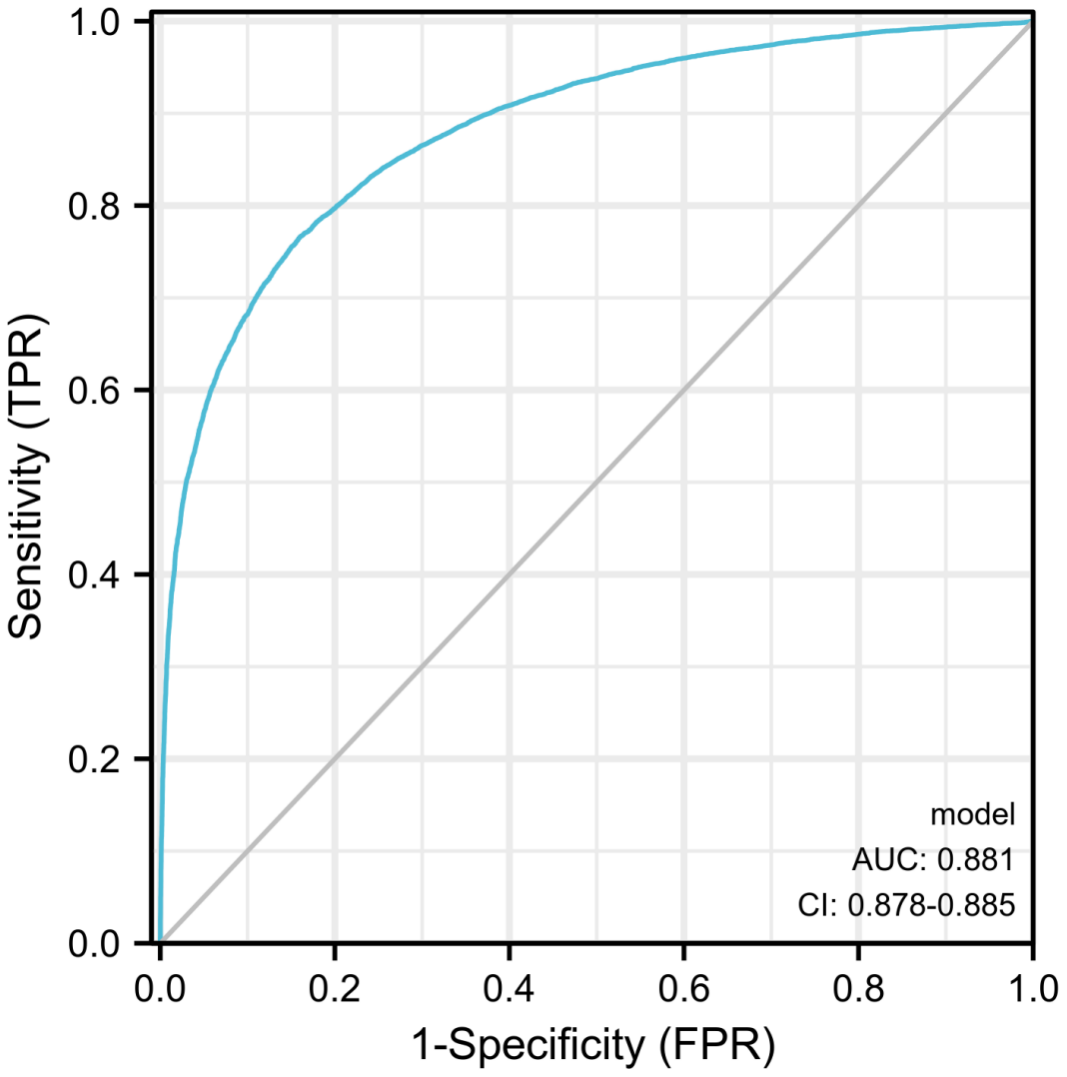
SBP, BMI, ALT, TG, and HDL indicators for a combined diagnostic ROC analysis curve.

Diagnostic ROC analysis of age, SBP, DBP, BMI, ALT, AST, TBIL, FBG, TC, TG, HDL, and LDL indicators showed an accuracy of prediction for SBP (AUC = 0.708, CI = 0.702–0.714) with a cut-off value of 125.50. The prediction of BMI was accurate (AUC = 0.836, CI = 0.832–0.841) with a cut-off value of 23.25. The prediction of ALT was accurate (AUC = 0.767, CI = 0.761–0.772) with a cut-off value of 16.50. The prediction of TG was accurate (AUC = 0.780, CI = 0.775–0.785) with a cut-off value of 1.385. HDL was predicted with accuracy (AUC = 0.723, CI = 0.718–0.729) with a cut-off value of 1.315. The accuracy of the predictive ability of the remaining indicators was poor (Figure 2; Table 3). We performed a combined diagnostic ROC analysis of SBP, BMI, ALT, TG, and HDL indicators with predictive power, and the results showed that the combined analysis of these indicators was accurate for the diagnosis of NAFLD (AUC = 0.881, CI = 0.878–0.885), as shown in Figure 3.

**Table 3:** Analysis of relevant parameters under the best cut-off value of each index of the ROC curve.

	Cut-off	Sensitivity	Specificity	Positive predictive	Negative predictive	Yoden index
Age	46.5	0.550	0.719	0.630	0.648	0.269
SBP	125.500	0.610	0.714	0.650	0.678	0.325
DBP	78.500	0.567	0.677	0.604	0.643	0.245
BMI	23.250	0.702	0.817	0.769	0.759	0.519
ALT	16.500	0.669	0.731	0.684	0.718	0.400
AST	19.500	0.558	0.681	0.604	0.640	0.240
TBIL	10.850	0.322	0.708	0.489	0.546	0.030
FBG	5.350	0.680	0.600	0.596	0.683	0.280
TC	4.995	0.565	0.559	0.527	0.596	0.123
TG	1.385	0.671	0.750	0.700	0.724	0.421
HDL	1.315	0.627	0.708	0.652	0.686	0.336
LDL	2.985	0.597	0.538	0.529	0.605	0.135

## 4. Discussion

In this study, we analyzed the health data of 31,718 patients to study the risk factors associated with the occurrence of NAFLD. We found that age, SBP, DBP, BMI, ALT, TBIL, FBG, TG, and LDL were risk factors for the occurrence of NAFLD and age, BMI, ALT, TG, and HDL were valuable for diagnosing NAFLD. We also performed a combined analysis of several indicators with an AUC area above 0.7 and found that the combined five indicators of age, BMI, ALT, TG, and HDL had diagnostic value for the diagnosis of NAFLD (AUC = 0.881).

Here we found age a risk factor for NAFLD development. The results of multifactorial logistic regression analysis showed that the risk of NAFLD increased by 2.9% for each year of age, which is consistent with the results of several studies (7–11). The results of the ROC analysis showed a cut-off value of 46.5 years, which suggests that middle-aged and adults patients should be more aware of and prevent the occurrence of NAFLD. The liver’s structure and function change considerably with aging, and many of the liver’s metabolic and detoxifying active functions change over time, gradually disturbing the body’s homeostasis and leading to functional decline (12). As a result, the adults are more prone to diseases such as cancer, cardiovascular disease, hypertension, diabetes, and hyperlipidemia (13), which may be associated with the development of NAFLD. Compared to younger individuals, older adults have lost nearly one-third of their liver volume and blood perfusion, which may adversely affect the regenerative capacity of their liver (14). In addition to age-related lipid accumulation in non-adipose tissues (including the liver), old age is associated with bone loss, decreased muscle mass and function, and dysregulation of free radical scavenging systems, which may lead to increased oxidative stress, all of which contribute to the progression of NAFLD (15).

ALT is a common indicator of liver function. According to our study, ALT was an independent risk factor for the development of NAFLD (OR = 1.059,95% CI: 1.054–1.063), which is consistent with several other studies (16, 17), and ALT has a diagnostic value for NAFLD (AUC = 0.767) with a cut-off value of 16.5. However, the use of ALT for the diagnosis of NAFLD is controversial because it is not possible to determine the sequence of NAFLD occurrence and abnormal ALT levels. Several studies have shown that NAFLD or NASH is present in a subset of the population, despite normal transaminases (18, 19). A recent study also pointed out that elevated ALT, AST, and γ-GT are not reliable markers of NASH or progressive NAFLD. Likewise, serum concentrations of these factors within the normal range do not exclude NAFLD. However, when NAFLD is diagnosed by other methods, transaminase levels can still be used for disease monitoring (20).

Our study also identified SBP, DBP, BMI, FBG, TG, and LDL as risk factors for the development of NAFLD. Abnormalities in these indicators corresponded to each of the components included in METS. METS usually includes obesity, hyperglycemia, dyslipidemia, and hypertension (21). Some studies have pointed out that METS is the strongest risk factor for NAFLD and NASH (3). NAFLD and METS may be mutually influential factors, especially in terms of hyperglycemia and hypertension. METS not only increases the risk of NAFLD, but NAFLD may enhance some features and complications of METS. Therefore, the treatment of NAFLD may improve METS. METS is also an important influencing factor for adverse cardiovascular events and mortality in patients with NAFLD (22, 23).

Obesity is a key factor in the development of NAFLD. In the present study, we found that BMI was a risk factor (OR = 1.413, 95% CI: 1.395–1.431) and the best predictor (AUC = 0.836) of NAFLD, which is in accordance with several other studies (24, 25). Recent studies have shown that obesity is strongly associated with NAFLD and liver fibrosis (26). The prevailing theory regarding its causes is the “spillover hypothesis,” which points out that subcutaneous tissues have a limited capacity to carry the size and number of adipocytes. When excess fat leads to excess subcutaneous fat, lipids will accumulate in other less adaptable tissues, especially the liver, leading to NAFLD (27). In the long term, imbalance in lipid metabolism leads to the excessive formation of toxic intermediates, which can lead to cellular stress (i.e., oxidative stress and endoplasmic reticulum stress), inflammatory vesicle activation, and apoptotic cell death, followed by inflammation, tissue regeneration, and fibrosis (3).

Dyslipidemia is also a risk factor for the development of NAFLD. In this study, LDL was the strongest risk factor for NAFLD. The risk of NAFLD increased 1.285 times for each unit increase in LDL level. TG has diagnostic value for NAFLD by a ROC analysis study (AUC = 0.78). The pathogenesis of NAFLD is thought to be related to hepatocellular fat accumulation and dysregulation of fatty acid metabolism, leading to steatosis, as well as hepatocyte inflammation and necrosis (28). In conclusion, lipid levels are closely related to the development of NAFLD.

Among the characteristics of METS, hyperglycemia is most clearly biologically linked to the progression of NAFLD, with up to 75% of patients with type 2 diabetes suffering from NAFLD. Patients with NAFLD who have diabetes also have a higher prevalence of NASH and advanced fibrosis compared to non-diabetic patients with NAFLD, and a higher likelihood of liver injury, regardless of elevated blood transaminase levels (29–31). These studies all suggest that high glucose status is strongly associated with the development of NAFLD, likely because it promotes hepatic lipid accumulation, increased lipotoxicity, liver injury, and inflammation.

Hypertension is one of the most common chronic diseases today, and it has a close relationship with several diseases. This study found hypertension as one of the risk factors for NAFLD. Approximately 50% of hypertensive patients have NAFLD (32), and NAFLD is associated with changes in arterial stiffness, myocardial remodeling, renal disease, and heart failure (33–35). In an Italian cohort study of patients with NAFLD, those with hypertension had a higher risk of liver fibrosis progression during a 6.2-year follow-up period (36). A recent study (37) showed a higher prevalence of NAFLD in patients with metabolic dysfunction, such as hyperglycemia, dyslipidemia, and hypertension, with a prevalence of 38.5% in the hypertensive group and 12.8% in the non-hypertensive group.

In our study, two indicators, AST and TC, caught our attention. The results of single-factor logistic regression analysis showed that both indicators were risk factors for the occurrence of NAFLD, but when accessing multi-factor logistic regression analysis, both became protective factors. We speculate that this may be due to the influence of other risk factors on these two indicators. Compared to other stronger risk factors, the effects of AST and TC on NAFLD were relatively weak, which is why the above two factors eventually became protective factors after the multifactor logistic regression analysis was performed.

In conclusion, we analyzed the medical examination information of some residents in Qingpu, Shanghai, and the results showed that the occurrence of NAFLD is closely related to age, METs, and other indices. Individuals should manage their BMI, blood glucose, hypertension, and blood lipids, and monitor liver function indexes, to prevent the occurrence of NAFLD.

## Data availability statement

The raw data supporting the conclusions of this article will be made available by the authors, without undue reservation.

## Ethics statement

This study was approved by the Ethics Committee of Qingpu Branch of Zhongshan Hospital, Fudan University, Shanghai, China, and was conducted in accordance with the principles outlined in the Declaration of Helsinki. The patients/participants provided their written informed consent to participate in this study.

## Author contributions

GW, XS, and YW wrote the manuscript and conceived and designed the manuscript. HL and XW analyzed the data. HH and XW collected and provided the sample for this study. All authors contributed to the article and approved the submitted version.

## Funding

This study was supported by Shanghai Natural Science Foundation (20ZR1411900), Shanghai Health Care Commission (202040065), Research Project of Qingpu Branch of Zhongshan Hospital, Fudan University (QYM2020-06), the Scientific Research Foundation provided by Pudong Hospital affiliated to Fudan University (project nos. Zdxk2020-01, Zdzk2020-09, and YJYJRC202104), Fudan Zhangjiang Clinical Medicine Innovation Fund Project (KP7202105), and the Pudong New Area Clinical Characteristic Discipline Project (grant no. PWYts2021-11).

## Conflict of interest

The authors declare that the research was conducted in the absence of any commercial or financial relationships that could be construed as a potential conflict of interest.

## Publisher’s note

All claims expressed in this article are solely those of the authors and do not necessarily represent those of their affiliated organizations, or those of the publisher, the editors and the reviewers. Any product that may be evaluated in this article, or claim that may be made by its manufacturer, is not guaranteed or endorsed by the publisher.
